# Heat shock protein gene expression varies among populations but does not strongly track recent environmental conditions: implications for biomarker development

**DOI:** 10.3389/fphys.2025.1601369

**Published:** 2025-10-23

**Authors:** Mary J. Woodruff, Sarah E. Wolf, Ethan D. Clotfelter, Elizabeth P. Derryberry, Mark T. Stanback, Kimberly A. Rosvall

**Affiliations:** ^1^ Department of Biology, Indiana University, Bloomington, IN, United States; ^2^ Center for the Integrative Study of Animal Behavior, Indiana University, Bloomington, IN, United States; ^3^ Center for Epidemiology and Animal Health, US Department of Agriculture Animal and Plant Health Inspection Service, Fort Collins, CO, United States; ^4^ Institute of Ecology and Evolution, School of Biological Sciences, University of Edinburgh, Edinburgh, United Kingdom; ^5^ Department of Biology, Amherst College, Amherst, MA, United States; ^6^ Department of Ecology and Evolutionary Biology, University of Tennessee Knoxville, Knoxville, TN, United States; ^7^ Department of Biology, Davidson College, Davidson, NC, United States

**Keywords:** heat shock protein, biomarker, thermal tolerance, populations, bird

## Abstract

**Introduction:**

Global temperatures are rising, and scientists are mobilizing to uncover which birds are most affected by the problem of heat. Heat shock proteins (HSPs), for example, can shed light on this issue because they prevent damage and promote recovery from heat. However, few studies have investigated the relationship between HSPs and heat outside of experimental contexts. Here, we ask whether natural variation in HSP gene expression can serve as a biomarker of recent ambient conditions in wild nestling tree swallows (*Tachycineta bicolor*).

**Methods:**

We focused on HSP90AA1 because this HSP increases mRNA abundance in avian blood, after acute heat. Using blood samples collected across ten degrees of latitude, we tested for population differences in constitutive HSP90AA1 gene expression in 12-day-old nestlings. To quantify the specific time period over which ambient conditions best predicted variation in HSP gene expression, we used a climate window analysis, evaluating the predictive value of maximum temperatures and maximum heat index in the hours and days from hatching until sampling.

**Results:**

We observed a significant difference in constitutive HSP gene expression between populations, with South Carolina nestlings showing nearly double the HSP90AA1 mRNA abundance compared to those in Massachusetts. There was no relationship between HSP90AA1 and heat index at any time (hours or days), meaning that baseline HSP gene expression is not a reliable biomarker for the combined effects of heat and humidity, at least not when applying existing metrics that were developed for poultry. We found some evidence linking HSP90AA1 gene expression with maximum temperatures three to four days before sampling; however, a permutation test could not rule out the possibility of a false positive.

**Discussion:**

HSP90AA1 mRNA abundance is not necessarily an effective biomarker of recent heat, and it may instead reflect other inherent population differences. As heat waves intensify, this conclusion could change, and other species could be more reactive to heat. We urge the avian biology community to continue biomarker testing for estimating heat impacts on wild birds, as we seek to better understand and predict avian resilience to environmental challenges.

## Introduction

Anthropogenic change is driving temperature increases across the globe (Fischer et al., 2021). In extreme cases, high temperatures can cause population die-offs (Kim and Stephen, 2018; Mckechnie and Wolf, 2019) or species extinction (Root et al., 2003). Less intense, sub-lethal, heat can still change animal behavior and physiology (Louis et al., 2020; Woodruff et al., 2023). However, the impacts of sublethal heat are challenging to measure in natural environments because thermally-sensitive phenotypes can reflect an individual’s recent heat exposure as well as an individual’s readiness to cope with future heat (discussed in Svensson et al., 2023), complicating interpretation of data (Kenkel et al., 2014). Amidst the challenges of quantifying heat effects in wild animals, there is a real need to develop and test biomarkers *in situ*, particularly those that can be applied to multiple species by multiple investigators (Kenkel et al., 2014) to monitor an organism’s recent exposure to some environmental agent (Califf, 2018). Most biomarker testing and development focuses on human health and disease (Califf, 2018), but there is a growing arm of conservation physiology, which uses elements of stress physiology in wild animals to measure anthropogenic impacts (Beaulieu and Costantini, 2014; Dantzer et al., 2014), including those caused by thermal stress (Parkinson et al., 2020; Sejian et al., 2018).

Much of this work has focused on mammals (Madliger et al., 2018) or aquatic ectotherms (Fangue et al., 2006; Kenkel and Matz, 2016; Li et al., 2019), but research on birds and the problem of heat has lagged behind, until the last 5–10 years (Mckechnie and Wolf, 2019; Nord and Giroud, 2020). Early work on avian thermal tolerance understandably related to poultry science, considering that heat stress is an economic issue for meat and egg production (reviewed in: Etches et al., 2008; see also; Greene et al., 2019; Kang and Shim, 2021; Murugesan et al., 2017; Nyoni et al., 2019; Wan et al., 2017; Wang et al., 2013; Xie et al., 2014; Zulovich and Deshazer, 1990). In recent years, this line of research has broadened to more bird species in more environments, including both laboratory and field (Andreasson et al., 2018; Andrew et al., 2017; Choy et al., 2021; Corregidor-Castro and Jones, 2021; Mckechnie et al., 2021; Pollock et al., 2021; Rodriguez and Barba, 2016; Ton et al., 2021; Woodruff et al., 2023). With this broader foundation of knowledge on the diverse physiological mechanisms that respond to heat in birds (Hoffman et al., 2018; Hsu et al., 2020; Lipshutz et al., 2022; Mentesana and Hau, 2022; Woodruff et al., 2025), we are well positioned to explore biomarkers of thermal tolerance with which we might predict resilience to future climate conditions.

Heat shock protein (HSP) regulation is one physiological metric that has potential as a biomarker of heat tolerance (Corbett et al., 2023; Greene et al., 2019; Hoffman et al., 2024). HSPs are a protective response to heat and other stressors, and they prevent cellular damage and promote recovery (Feder and Hofmann, 1999; Lindquist and Craig, 1988; Singh et al., 2024). After direct heat exposure, HSP gene expression peaks approximately 4 hours later, though HSPs represent a large gene family and exact timing varies by gene (Finger et al., 2018; Foster et al., 2015). Gene expression can also remain elevated above baseline levels 24 h later (Wan et al., 2017), suggesting lingering effects on constitutive HSP expression (i.e., levels in the absence of acute heat). Species, populations, or breeds from warmer climates can show higher levels of HSP gene expression, even when exposed to the same thermal regimes (Fangue et al., 2006; Singh et al., 2014; Wan et al., 2017; Xie et al., 2018). Having a higher baseline of HSP expression could counteract heat-induced damage as soon as it begins, potentially negating the need for further elevation when faced with subsequent heat (Gleason and Burton, 2015; Kenkel and Matz, 2016; Li et al., 2019). While this provides promising evidence that HSPs may be a biomarker of thermal exposure or thermal tolerance, little work has examined the degree to which baseline HSP levels may reflect inherent biological differences among populations versus a response to recent temperatures that *happen* to differ among populations. The time course of HSP elevation can vary over evolutionary time (Li et al., 2019; Tomanek and Somero, 2000), meaning it is critical that we determine the degree to which recent ambient conditions influence presumed baseline levels of HSPs, in birds.

Here we investigate the degree to which recent environmental conditions predict constitutive (naturally-occurring) blood HSP gene expression in nestling tree swallows (*Tachycineta bicolor*), across geographically distinct populations. We aimed to identify critical time windows during early life when ambient conditions might predict HSP levels, and if so, we sought to determine the timescale over which these effects are integrated, from hours to days to weeks. Specifically, we tested three non-mutually exclusive hypotheses: (1) HSP expression reflects very recent conditions in the hours before sampling, (2) HSP expression integrates environmental experience during critical developmental time windows (e.g., hatching or peak growth), or (3) HSP expression reflects evolved population-level differences across thermal regimes, independent of recent environmental exposure. Examining these predictors of thermal physiology is critical for working towards a much-needed biomarker for heat tolerance in free-living birds.

## Materials and methods

### Study sites and sampling design

**FIGURE 1:**
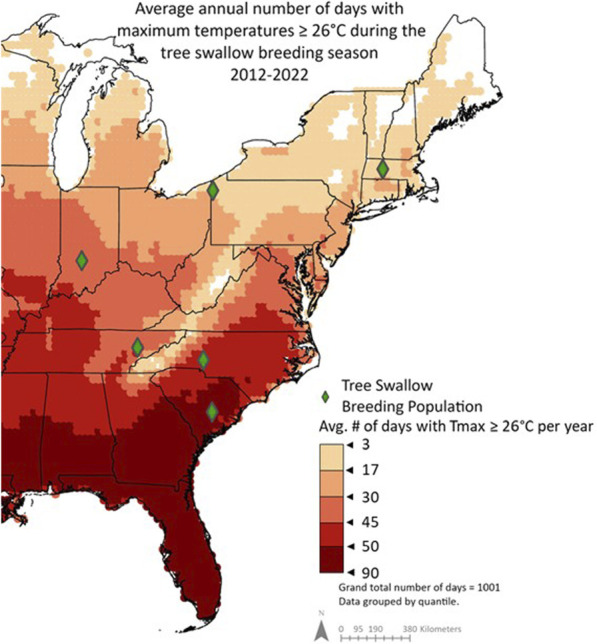
Average annual number of days during the tree swallow breeding season (April-June) with maximum temperatures ≥26 °C from 2012-2022. Previous research demonstrated that nestlings begin thermoregulating when nest temperatures reach 38 °C–equivalent to 26 °C air temperatures because nestboxes are generally 12 °C hotter than ambient (Woodruff et al., 2023). Therefore, days with maximum air temperatures ≥26 °C were included in this summary. Data is grouped by quantile.

Our study focused on nestling tree swallows confined to the thermal environment of a nesting cavity—here, a human-made nest box. Further, the tree swallow breeding range spans much of North America (Winkler et al., 2020), allowing us to sample geographically distinct populations. We collected data from six populations, encompassing ∼10 degrees of latitude across the eastern United States, from South Carolina to Massachusetts (Table 1; Figure 1). While these populations extend to nearly the southern end of the species breeding range (Mccaslin and Heath, 2020; Shutler et al., 2012), our sampling does not capture the northern half of the breeding range, namely, Canada and Alaska (Winkler et al., 2020). We targeted sampling to postnatal day 12 (D12) because nestlings are fully endothermic and have reached asymptotic, adult-like mass (Mccarty, 2001), but they are young enough that researcher visits do not risk fledging, which occurs around postnatal D21. Hatch day is denoted as D1, and represents the day the majority of nestlings hatched, though it is noteworthy that there is marked hatching asynchrony in tree swallows (Winkler et al., 2020), so some of the sampled chicks may have hatched a day earlier. Prior to hatching, eggs are incubated by mothers for approximately 12 days and therefore are kept at a relatively consistent temperature via maternal modulation of incubation (Coe et al., 2015; Huggins, 1941). From D1 to D6 post-hatch, nestlings are ectothermic (Marsh, 1980) and are brooded by mothers; after this point, they are more exposed to ambient temperatures and can display thermoregulatory behaviors, such as panting and huddling (Woodruff et al., 2023).

**TABLE 1:** Study populations name and location, sample size, year samples collected, and NOAA weather station name and location. We collected blood samples from one nestling per nest, therefore sample size values reflect unique nests and nestlings. See details in SI§ B.

State	State Abbrev.	County	Latitude (°N)	Longitude (°W)	N	Year(s) sampled	NOAA weather station	Weather station latitude (°N)	Weather station longitude (°W)	Distance to study area
Massachusetts	MA	Hampshire	42.22	72.31	14	2020	Hartford Bradley Airport	41.94	72.68	50 Km
Pennsylvania	PA	Crawford	41.65	80.43	36	2020	Port Meadville Airport	41.64	80.23	18 Km
Indiana	IN	Monroe, Brown	39.17	86.53	20	2019	Monroe County Airport	39.14	86.62	14 Km
Tennessee	TN	Knox	35.9	83.96	30	2020	Knoxville Airport	35.82	83.99	11 Km
North Carolina	NC	Iredell	35.53	80.88	14	2020	Statesville Municipal Airport	35.77	80.96	28 Km
South Carolina	SC	Clarendon	33.49	80.36	33	2020, 2021	Charleston Airport	32.9	80.04	45 Km

### Blood sampling and nestling age estimation

We collected blood at 25 ± 4 nests per population (Table 1) when nestlings were 11.9 ± 0.1 days-post-hatch (range: D9 – D15). We sampled 2-3 nestlings per nest, avoiding obvious runts and bleeding from the alar vein (∼50 uL). Samples were collected around mid-day (average 12 h 10m ± 0 h 08m) during the summer breeding seasons of 2019, 2020, and 2021. Blood was stored on dry ice in the field and later transferred to −80 °C freezer. We banded all nestlings with a numbered USGS band, measured body mass to the nearest 0.1 g, and measured wing length to the nearest 0.5 mm. For most nests, we knew the exact hatch date. However, we estimated some hatch dates in Pennsylvania, Tennessee, North Carolina, and South Carolina, due to pandemic-related personnel constraints. When hatch date was not known exactly, we estimated age using published tree swallow growth trajectories (Mccarty, 2001; Wolf et al., 2021); details in *SI§ A*. Nestling mass and days-post-hatch when sampled had no effect on HSP90AA1 gene expression (details in *SI§* *A*).

Later, we measured HSP gene expression from the median mass nestling. In nests where only two nestlings were sampled, we randomly selected one for analysis. Sample sizes across populations differ due in part to pandemic-related personnel constraints, RNA quality, and weather conditions (details in *SI§* *A*).

### Environmental data and heat index calculation

We collected environmental data from two types of long-term databases to evaluate the degree to which populations differed in thermal regimes during breeding.

First, to assess population differences in breeding season climates, we used 10 years of remote-sensing data (ERA5 hourly data, Hersbach et al., 2022) to quantify the number of days in which nestling tree swallows were likely to experience sub-lethal heat. Specifically, we focused on days with environmental temperatures above the estimated thermoneutral zone, a range of ambient temperatures outside of which an animal exerts energy to regulate internal temperatures, during the typical nestling season. The exact upper limit of this “comfort” zone is not known, but at least two types of inferences suggest it occurs around 38 °C (details in *SI§ B*). If we account for the observation that nest cup average temperature was 12.3 °C ± 0.8 °C warmer than the ambient temperature (Woodruff et al., 2025), then nestlings have the potential for sublethal heat stress at ambient temperatures of about 26 °C. We then quantified the number of days during which maximum ambient temperatures met or exceeded 26 °C (air temperature 2 m above land surface, resolution 0.1° × 0.1° from Copernicus Climate Change Service Climate Data Store; Hersbach et al., 2022). Based on our experience in these populations and typical lay dates, we focused on April 1st–June 30th, n = 91 days/year from 2012-2022. We then mapped these data using ArcGIS Pro (version 3.0.2); Figure 1.

Second, to collect climate data with which to predict HSP gene expression, we downloaded hourly dry bulb and wet bulb temperature data from the National Oceanic and Atmospheric Administration (NOAA) weather station nearest to each field site (Table 1). Wet bulb temperatures can be used to calculate humidity and therefore heat index. High temperatures are known to affect HSP gene expression (e.g., Fangue et al., 2006), but humidity may affect the experience of a temperature and the effectiveness of thermoregulation (Gerson et al., 2014; Van Dyk et al., 2019). Therefore, a heat index can be helpful for understanding the experience of heat since it combines the effects of temperature and humidity into one value. Unfortunately, to our knowledge, there is no heat index formula for estimating effective heat for songbirds. Therefore, we used a physiologically-based index that was originally designed for laying hens, in Zulovich and Deshazer (1990); Supplementary Formula S1. We chose this formula because among the available indices, laying hens are the closest in physical size to tree swallows and therefore may best estimate effective heat. We used hourly dry bulb and wet bulb temperatures to calculate hourly heat index. In the end, our analyses focus on daily maximum values for temperature (T_max_) and heat index (Heat Index_max_).

### Gene expression (qPCR)

We quantified relative gene expression using RNA extracted from blood. Briefly, we extracted RNA using Trizol and converted RNA to cDNA using Superscript III (details in *SI§ C*). We then ran cDNA in triplicate in quantitative real-time PCR (qPCR) to measure mRNA abundance of HSP90AA1, a gene that is robustly linked to heat tolerance in birds (Wang et al., 2015). Our previous work on HSP90AA1 in nestling tree swallows shows that it is expressed abundantly in blood, and its expression is elevated approximately 2-fold after a 4-h experimental heat challenge (Woodruff et al., 2025). Therefore, we focused on HSP90AA1 gene expression in this study because it should be reactive if nestlings were responding to a heat challenge. mRNA abundance was calculated in ThermoCloud (Thermo Scientific) using the delta Ct method in which fold change in expression for the gene of interest is normalized to an internal reference gene, MRPS25 (2^−Δ∆Ct^, where ∆ΔC_t_ = (C_t_^HSP90AA1^ – C_t_^MRPS25^) _reference_ – (C_t_^HSP90AA1^ – C_t_^MRPS25^) _sample_). Details on qPCR methods are in *SI§ C* and Supplementary Table S3. Plates were balanced by population and date. Each plate included intra- and inter-plate control samples (a cDNA pool derived from tree swallow RNA) and the ThermoCloud used these samples to normalize values across plates. We found no significant effect of sex on HSP90AA1 gene expression; therefore, we did not include sex a covariate in subsequent analyses (details in *SI§* *C*).

### Statistical analyses

All analyses were performed in R Studio (2022.07.2 Build 576), and HSP90AA1 relative quantities were Log2 transformed to improve normality. We conducted two types of analyses: First, we explored main effects of population on environmental variables and HSP gene expression. Second, we explored co-variation between HSP gene expression and environmental variation. To assess whether temperature extremes may shape the nature of this co-variation, we conducted this latter analyses for all populations (considered together) and again for South Carolina only (the southern-most population). For all analyses, we ensured that variables were not multicollinear (all VIFs <3, as in Fox and Weisberg, 2018).

#### Testing for main effects of population

We assessed population differences in environmental data (T_max_ and HeatIndex_max_), averaged across two time periods: (1) from hatching to sampling and (2) during the 4 hours preceding sampling. We used linear mixed effects models in which the climate variable was predicted by population. Because some nestlings were sampled on the same day, we included the random effect of Julian date. We assessed population differences in HSP90AA1 gene expression via ANOVA with a fixed effect of population. Pairwise comparisons were analyzed in a *post hoc* Tukey test.

#### Testing for co-variation between environment and gene expression

We assessed whether and how environments shape constitutive HSP gene expression with a sliding window analysis using the “slidingwin” function in the *climwin* R package (Van De Pol et al., 2016). This function is designed to identify time periods or “windows” over which a biological variable is sensitive to environmental variables (Bailey and Van De Pol, 2020). Climate windows varied in length from 1 day to 13 days, corresponding to the nestlings’ entire post-hatch life from est. hatch day up until sampling day (treated as day 0 by the program). We used all combinations of window start and window end days during this time. For example, “window open = 1, window close = 1” corresponds to ambient conditions only on the 1 day before sampling. At the other extreme, “window open = 13, window close = 1” corresponds to ambient conditions averaged across the 13 days leading up to sampling. This window is wider than 12 days (i.e., the estimated age at sampling) to accommodate the aforementioned potential for intra- or inter-nest variability in hatching; further details in *SI§* *D*.

For each climate window, we derived the mean value of our daily T_max_ and HeatIndex_max_ data. We then fit a linear model for Log2 HSP90AA1 relative quantity with population and the climate window variable as predictors. The ‘null’ model consisted of Log2 HSP90AA1 relative quantity predicted by population alone, i.e., it modeled the situation in which gene expression varies among populations regardless of recent ambient conditions.

Because blood samples were collected at midday, the maximum temperature of the sampling day may have occurred *after* the sample was collected, such that “window open = 0, window close = 0” is not applicable. The sliding window package does not allow for temperature windows to be tailored to a specific number of *hours*, so we used a separate linear model to ask if “day-of” environments affected HSP gene expression. Specifically, we used maximum environmental variables from the 4 hours before sampling. We also verified that the maximum temperature nestlings experienced on the day of sampling (12a.m.–time of sampling) occurred during the 4 hours proceeding sample collection.

Finally, for each climate variable, we merged results from the sliding window and manual “day of” models and compared them with Akaike’s information criterion adjusted for small sample sizes (AICc). We considered models with ΔAICc ≤2 from ‘null’ to be competitive and equally well-fit (Burnham and Anderson, 2002). Given the number of models in this analysis, type 1 errors are possible (Van De Pol et al., 2016). Therefore, we also tested the likelihood of obtaining a similar ΔAICc using randomized climate variables as in Van De Pol et al. (2016). This step uses the ‘randwin’ function in *climwin* to generate 1,000 randomized models and a probability of getting our results by chance (hereafter PΔAICc). Climate variables for which the observed AICc of the best fit model was different from the randomized results (PΔAICc <0.05) indicate that the variable predicts HSP gene expression beyond what is likely to occur by chance alone. We repeated this same analysis for South Carolina alone because this southernmost population captures one of the warmest possible early life climates for the species (Mccaslin and Heath, 2020; Shutler et al., 2012). We did not have the power to replicate this analysis in the northern populations because personnel constraints limited the number of days during which blood samples were collected, thus restricting the amount of potential explanatory ambient conditions.

## Results

### Population differences in environmental variables

Examining a decade of climate data, we found that our study populations differed in potential exposure to sublethal heat, from an average of 3–90 days per year (Figure 1). During the study period, we observed significant population differences in mean daily T_max_ from hatching to sampling (F_5,136.4_ = 35.76, p < 0.001) and T_max_ in the 4 hours prior to sampling (F_5, 131.08_ = 11.44, p < 0.001); summarized in Table 2. Similarly, we observed significant population differences in mean daily HeatIndex_max_ from hatching to sampling (F_5, 136.00_ = 41.30, p < 0.001), and HeatIndex_max_ in the 4 hours prior to sampling (F_5,129.45_ = 9.79, p < 0.001). Post-hoc Tukey tests showed generally lower T_max_ and HeatIndex_max_ in more northern populations (Massachusetts and Pennsylvania) compared to South Carolina across time periods (Table 2).

**TABLE 2:** Environmental variable mean ± standard error and range by population. For nestling period values, daily *T*_max_ and *HeatIndex*_*max*_ were averaged per nest from hatching to sampling, then the mean values were summarized per population. Letters indicate pairwise comparisons resulting from a *post hoc* Tukey test based on a linear mixed effects model that controlled for the random effect of Julian date.

Environmental variable	State	Mean ± SE	Range	Tukey test pairwise comparisons
Mean T_max_ Nestling Period (°C)	MA	26.58 ± 0.02	26.43–26.60	ab
	PA	26.24 ± 0.06	25.58–26.60	b
	IN	27.02 ± 0.31	24.91–31.50	b
	TN	26.77 ± 0.73	20.64–32.95	a
	NC	22.54 ± 0.45*	20.87–24.17	c
	SC	26.19 ± 0.40	20.64–29.79	d
T_max_ 4 h Prior (°C)	MA	24.47 ± 0.51	21.11–27.78	ab
	PA	23.24 ± 0.55	17.22–29.44	a
	IN	25.94 ± 0.63	19.44–31.11	ab
	TN	26.94 ± 0.58	18.33–31.94	bc
	NC	27.28 ± 0.60	23.52–30.00	c
	SC	28.56 ± 0.36	23.89–30.56	c
Mean HeatIndex_max_ Nestling Period	MA	23.06 ± 0.02	23.18–22.95	a
	PA	22.81 ± 0.05	23.18–22.30	a
	IN	24.70 ± 0.29	29.05–22.83	b
	TN	23.56 ± 0.69	29.13–17.55	C
	NC	19.49 ± 0.45	21.15–17.79	d
	SC	22.86 ± 0.37	26.09–17.55	b
HeatIndex_max_ 4 h Prior	MA	21.16 ± 0.54	15.62–24.67	a
	PA	20.22 ± 0.52	14.45–26.11	a
	IN	23.42 ± 0.66	16.55–27.78	b
	TN	23.92 ± 0.51	17.44–28.56	bc
	NC	24.45 ± 0.49	21.81–27.04	c
	SC	25.43 ± 0.36	21.22–27.67	bc

*NC temperatures do not include the 3-day cold snap because none of the nestling periods included in this study overlapped with the cold snap.

### Population differences in HSP gene expression

**FIGURE 2:**
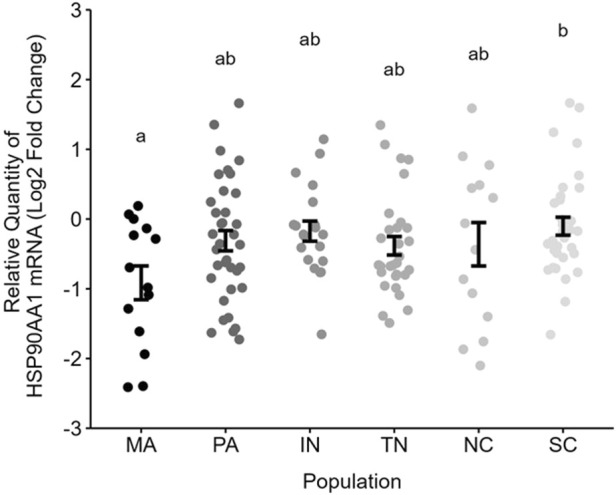
Relative gene expression of blood HSP90AA1 (Log_2_ 2^−ΔΔct^) across populations. Letters indicate pairwise comparisons resulting from a Tukey test. Each point represents one nestling per nest. Error bars are mean ± SE. Note that 1 unit is a 2-fold difference in abundance on this log2-scale.

We observed a marginal relationship between HSP90AA1 mRNA abundance and population (F_5, 141_ = 2.08, p = 0.07, *R* ^2^ = 0.07). A *post hoc* Tukey test showed significantly lower HSP gene expression in Massachusetts compared to South Carolina (t_141_ = −3.09, p = 0.03), though there were no other significant pairwise comparisons (Figure 2).

### Effect of “day of sampling” conditions

T_max_ and HeatIndex_max_ in the 4 h before sampling did not significantly predict HSP90AA1 gene expression better than the null model (Table 3). Within the warmest population (South Carolina), the same result held (Table 3).

**TABLE 3:** Day of sampling environmental conditions model results. AICc values relative to the null model (ΔAICc), beta estimate effect sizes (β), standard error (SE), and model intercept are reported. Window openings represent time furthest from sampling and window closures represent time nearest to sampling.

Models: All populations	Window open	Window close	ΔAICc	β	SE	Model intercept
Log2 HSP90AA1 ∼ 1	NA	NA	0	−0.32	0.07	−0.32
Log2 HSP90AA1 ∼ Population	NA	NA	0.24	−0.91	0.22	−0.91
Log2 HSP90AA1 ∼ Population + HeatIndex_max_	4hrs	Hour of sampling	1.72	0.02	0.03	−1.38
Log2 HSP90AA1 ∼ Population + T_max_	4hrs	Hour of sampling	1.94	0.02	0.02	−1.35

Models: South Carolina						
Log2 HSP90AA1 ∼ T_max_	4hrs	Hour of sampling	0	0.11	0.06	−3.38
Log2 HSP90AA1 ∼ 1	NA	NA	1.12	−0.10	0.13	−0.10
Log2 HSP90AA1 ∼ HeatIndex_max_	4hrs	Hour of sampling	1.24	0.09	0.06	−2.50

### Effect of environmental conditions across the nestling period

**FIGURE 3:**
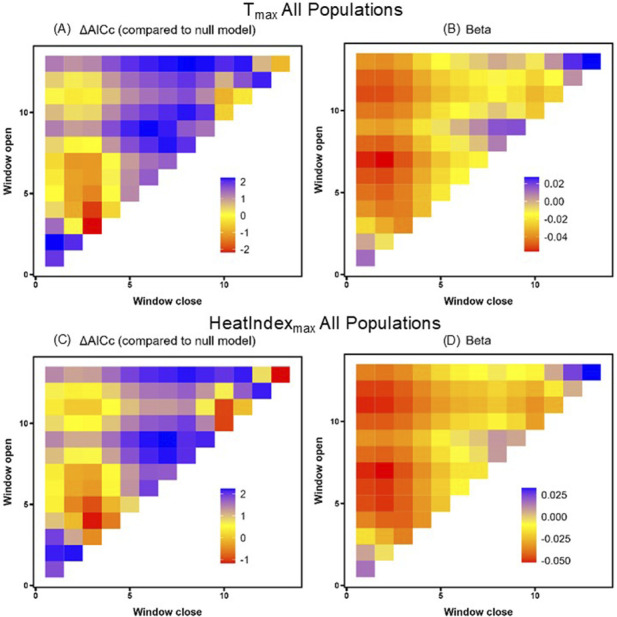
T_max_ **(A,B)** and HeatIndex_max_ **(C,D)** sliding window model ΔAICc values relative to the null model **(A,C)** and beta value effect sizes **(B,D)** for all populations. Window openings represent the time furthest from sampling and window closures represent the time nearest to sampling.

Using the sliding window analysis across all states, we found that T_max_ predicted HSP gene expression better than the null in two time windows. Specifically, the window from 3 days prior to sampling (open = 3, close = 3) and the window spanning three to 4 days prior to sampling (open = 4, close = 3) showed a negative relationship between HSP gene expression and T_max_ (Table 4; Figure 3), contrary to the positive co-variation we predicted. However, we could not reject the hypothesis that this result was a false positive (PΔAICc = 0.32). In the comparable model for HeatIndex_max_, the sliding window analysis found no windows predicting HSP gene expression better than the null (Table 4; Figure 3, PΔAICc = 0.67).

**TABLE 4:** AICc values relative to the null model (ΔAICc), beta estimate effect sizes (β), standard error (SE), and model intercept are reported. Window openings represent time furthest from sampling and window closures represent time nearest to sampling.

Models: All populations	Window open	Window close	ΔAICc	β	SE	Model intercept
Log2 HSP90AA1 ∼ Population + T_max_	3	3	−2.16	−0.04	0.26	0.05
	4	3	−2.01	−0.04	0.26	0.10
Log2 HSP90AA1 ∼ Population + HeatIndex_max_	13	13	−1.16	0.03	0.28	−1.77
	4	3	−1.14	−0.04	0.26	−0.05

Models: South Carolina						
Log2 HSP90AA1 ∼ T_max_	10	1	−4.92	−0.20	0.07	5.51
	10	2	−4.78	−0.17	0.06	4.77
	11	1	−4.48	−0.22	0.08	5.96
	11	2	−4.08	−0.18	0.07	5.02
	3	2	−4.00	−0.11	0.04	3.03
	9	2	−3.96	−0.15	0.06	4.03
	9	1	−3.83	−0.17	0.07	4.56
	8	2	−3.79	−0.14	0.06	3.86
	8	1	−3.47	−0.16	0.06	4.32
	3	3	−3.46	−0.11	0.04	2.86
	12	1	−3.40	−0.22	0.09	6.02
	8	8	−3.33	−0.08	0.03	2.11
	10	6	−3.07	−0.12	0.05	3.16
	4	2	−3.04	−0.13	0.06	3.60
	8	6	−3.02	−0.09	0.04	2.48
	8	7	−2.98	−0.09	0.04	2.31
	7	2	−2.88	−0.14	0.06	3.72
	12	2	−2.88	−0.18	0.08	4.93
	9	6	−2.76	−0.10	0.04	2.62
	10	7	−2.68	−0.11	0.05	3.03
	9	7	−2.52	−0.10	0.04	2.47
	10	3	−2.43	−0.13	0.06	3.48
	7	6	−2.43	−0.09	0.04	2.50
	9	8	−2.41	−0.09	0.04	2.40
	10	8	−2.39	−0.11	0.05	3.03
	7	1	−2.34	−0.15	0.07	4.06
	13	1	−2.30	−0.21	0.10	5.81
	6	6	−2.15	−0.09	0.04	2.39
	7	7	−2.11	−0.09	0.04	2.31
Log2 HSP90AA1 ∼ HeatIndex_max_	3	3	−4.71	−0.19	0.07	4.73
	3	2	−4.29	−0.17	0.06	4.08
	10	2	−4.19	−0.21	0.08	4.99
	10	1	−3.94	−0.23	0.09	5.67
	4	2	−3.81	−0.19	0.08	4.69
	9	2	−3.66	−0.18	0.07	4.42
	8	2	−3.58	−0.18	0.07	4.36
	8	7	−3.38	−0.12	0.05	2.64
	8	8	−3.33	−0.11	0.05	2.48
	7	7	−3.27	−0.12	0.05	2.73
	9	1	−3.20	−0.20	0.08	4.92
	8	1	−2.93	−0.20	0.08	4.81
	11	2	−2.91	−0.20	0.09	4.79
	8	6	−2.86	−0.12	0.05	2.75
	7	2	−2.86	−0.18	0.08	4.32
	11	1	−2.80	−0.23	0.10	5.51
	9	7	−2.71	−0.12	0.05	2.69
	9	6	−2.53	−0.12	0.05	2.80
	10	6	−2.50	−0.13	0.06	3.13
	10	7	−2.42	−0.13	0.06	2.99
	7	6	−2.37	−0.12	0.05	2.78
	9	8	−2.13	−0.11	0.05	2.52

**FIGURE 4:**
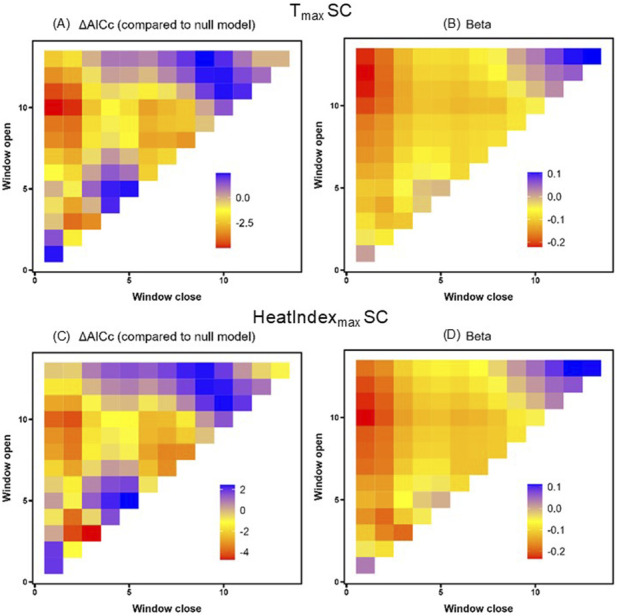
T_max_ **(A,B)** and HeatIndex_max_ **(C,D)** sliding window model ΔAICc values relative to the null **(A,C)** and beta value effect sizes **(B,D)** for South Carolina (SC). Window openings represent time furthest from sampling and window closures represent time nearest to sampling.

Finally, we focused on South Carolina alone, because its position at the southern extreme of the breeding range offered the warmest thermal environment for detecting potential effects. We again found that temperature was negatively related to HSP gene expression, with 29 windows spanning the entire nestling period in which T_max_ predicted HSP gene expression better than the null model (Table 4; Figure 4). Similarly, for HeatIndex_max_ we found 22 time windows that predicted HSP gene expression better than the null model, with a negative effect size in each window (Table 4; Figure 4). The direction of these results again did not align with our predictions, though neither of these results was robust to randomized validation (PΔAICc >0.33).

## Discussion

We measured naturally occurring variation in HSP90AA1 gene expression in wild nestling birds across ten degrees of latitude, including populations that differ in their environmental potential for heat stress (Figure 1) and their max temperatures experienced on the days we sampled (Table 2). HSP gene expression was ∼2-fold higher in the most southern population (South Carolina) compared to the most northern population in our study (Massachusetts). Other mid-latitude populations were not significantly different from one another. When we explored environmental predictors of HSP gene expression among nestlings across all populations, however, we found weak evidence connecting post-natal thermal temperatures with constitutive HSP gene expression. We also found no support for the hypothesis that HSP gene expression tracks heat index, at any time window preceding our sampling. One important caveat is that we used a humidity-informed heat index that was developed for poultry, and it will be important in the future to develop such an index for non-domesticated, wild birds. Though we found stronger evidence linking HSP gene expression and temperature in our southernmost (warmest) population, we could not reject the possibility of a false positive. Based on these collective results, HSP90AA1 gene expression in the blood is not a strong biomarker of recent exposure to heat, and it may more robustly reflect population-level differences in thermal physiology that have been shaped by long-term differences in climatic regimes (Figure 1). To the degree that these transcriptional patterns reflect protein abundance (Li and Biggin, 2015), we propose that HSP90AA1 mRNA abundance still has potential to reflect potentially adaptive readiness to handle heat, particularly as heat waves increase in their frequency and intensity.

### Population differences in 12 day-old nestlings

It is well established that physiology can vary among populations. For example, populations differ in baseline oxygen consumption (Storch et al., 2009), glucocorticoid secretion (Vitousek et al., 2019), telomere length (reviewed by: Burraco et al., 2020), and global gene expression (Gleason and Burton, 2015; Whitehead and Crawford, 2006). HSP90AA1, the focus of our study, is among those genes that have been linked to population differences, including in adult killifish (Fangue et al., 2006) and in domesticated sheep (Salces-Ortiz et al., 2015). Likewise, adult female tree swallows breeding in Alaska versus Indiana differed in HSP90AA1 gene expression in the hippocampus, with higher levels in the warmer lower latitude population (Woodruff et al., 2022). With the current study in nestlings, we extend this earlier finding to a much younger age (∼12 days-old), implying that differences in HSP gene expression from South Carolina to Massachusetts are “set” early in life, even if patterns also change with experience or age.

Mid-range latitudes did not differ in HSP gene expression, suggesting that tissue differences may also play a role here. Tissues have different naturally-occurring HSP gene expression levels (Woodruff et al., 2022) and are differentially impacted by heat (Leandro et al., 2004; Lipshutz et al., 2022). Blood has the logistical advantage that it can be sampled repeatedly, and in terms of biomarker development, blood may be the only possible tissue that can be sampled repeatedly and/or sampled in a threatened species. Previous research on nestling tree swallows demonstrated that HSP90AA1 gene expression in the blood is highly sensitive to experimental heat, more so than the brain or muscle (Woodruff et al., 2025), suggesting that it is unlikely that we are missing population differences simply by using blood.

Our climate analysis of the last decade shows that southern populations of tree swallows also have the potential to experience up to 30-times more warm days during the breeding season compared to the most northern populations we sampled (90 days vs. 3 days; Figure 1). Maximum heat index differed more among populations, but still by only about 5 °C. This limited environmental variation among populations may have occurred by chance, due to the logistics of sampling multiple populations. It also may relate to phenological adjustments because birds time their breeding to align with favorable conditions (De Villemereuil et al., 2020), with southern populations breeding before northern populations. Regardless, birds that breed earlier are at a higher risk of inclement weather (Shipley et al., 2020) and temperature extremes (Taff and Shipley, 2023), so there are likely to be constraints on advancing breeding too much.

If nestlings in South Carolina have more HSP90AA1 gene expression at their disposal during early critical periods of their development, what else may this reflect for them, organismally? Considering that HSPs prevent damage and promote recovery from heat (Feder and Hofmann, 1999; Lindquist and Craig, 1988), it is possible that higher baseline HSPs may reflect some degree of acclimation to challenging environments (Dong et al., 2008), hardening the organism to handle more heat in the future. Indeed, other species with higher baseline HSP expression may have less of a ‘need’ for further elevation in the face of heat (Kenkel and Matz, 2016; Li et al., 2019; Rinehart et al., 2006; Wan et al., 2017), but see (Fangue et al., 2006). Early life exposure could reduce the effects of subsequent heat on oxidative stress (Costantini et al., 2012) or reproduction (Hoffman et al., 2018), though there may be tradeoffs with other traits, such as immune function (Hoffman et al., 2018). More experimental work is needed to test reactivity to heat directly in the wild, particularly since acute heat may enhance among-individual differences in HSP gene expression (Woodruff et al., 2025) and HSP upregulation can be energetically costly (Sørensen, 2010).

### Effects of recent environmental conditions

To maximize the usefulness of transcriptomic biomarkers, it is paramount that we investigate what precisely we are measuring (Califf, 2018; Kenkel et al., 2014). Our sliding window analysis is an important step in this process because thermal conditions–across diverse timescales–can dramatically affect physiology (Gonzalez-Rivas et al., 2020; Mckechnie and Wolf, 2019).

Despite the documented reactivity of HSPs to heat within hours of exposure (Finger et al., 2018; Foster et al., 2015; Woodruff et al., 2025), we did not observe any significant relationships between same-day thermal conditions and HSP gene expression. Some time windows were associated with T_max_ variation across all samples, with more time windows seen in the South Carolina population; however, these patterns were not robust to false discovery (Table 4; Figure 4). Even if we try on these marginal effects for size, we note that all effect sizes in our top models were negative (refer to Table 4), meaning that the strongest relationships in these data link higher HSP gene expression with *lower*, not higher, temperatures. We speculate that this pattern may reflect downregulation of HSPs after a prior upregulation. The negative relationship between temperature and HSP gene expression also opposes the “response” or “reactivity” requirement for a biomarker (Califf, 2018) in which a biomarker should positively change in a meaningful way after exposure to a stimulus, though there are other reasons this may not rule out HSP90AA1 as a potential biomarker of recent heat exposure. For one, a null effect may occur if variation in HSP gene expression is also tracking other biotic or abiotic factors (Kaufmann, 1990; Lindquist and Craig, 1988), if HSP gene expression is shaped by parental effects on nestling phenotypes (Mota-Rojas et al., 2023), or if conditions were not extreme enough (Woodruff et al., 2023). Our analyses of the South Carolina samples aid interpreting this latter idea because these nestlings experienced the highest day-of-sampling temperatures in our study: on average, HeatIndex_max_ in South Carolina was 23 °C across the nestling period and max temperatures in the 4 hours preceding sampling as 29 °C. Based on our experimental data on nestling tree swallows in Indiana (Woodruff et al., 2025), this should have elevated HSP gene expression, yet our sliding window analysis did not link HSP gene expression to T_max_ beyond the posbility of false discovery. Pulling these inferences together, this means that our observation of higher HSP gene expression in South Carolina compared to Massachusetts cannot simply be a transient artifact of recent environmental conditions at the southern extreme of the species’ breeding range. We speculate that population-level differences in HSP90AA1 gene expression may better reflect ecological or evolutionary differences (sensu, Fangue et al., 2006; Singh et al., 2014; Wan et al., 2017). Thus, while HSPs are often thought of as a short-term response to recent heat, HSP levels measured outside of experimental contexts do not seem to be strong biomarkers of recent heat, unless that heat is more extreme than what we captured here.

Temperature alone may not tell the whole story because the compounded effects of heat and humidity can reduce the effectiveness of evaporative cooling (Gerson et al., 2014; Van Dyk et al., 2019). This is important for endotherms because evaporative cooling behaviors, like panting (Woodruff et al., 2023) or bathing (Oswald et al., 2008), are among the initial defenses against heat (Huey et al., 2012; Woodruff et al., 2023). A high heat index, which stems from high temperatures combined with high humidity, should therefore necessitate coping mechanisms beyond this initial (behavioral) front line, including HSP upregulation. Empirically, though, we did not support this expectation: we found no evidence linking HSP gene expression and maximum heat index. We used a heat index formula developed for birds, though it was developed for laying hens. While this was the best approximation available among the indices developed for birds (discussed in Purswell et al., 2012), it is reasonable to expect that a laying hen’s experience of heat may differ from that of a 20 g nestling. It is noteworthy that the NOAA heat index most people know is specifically designed to capture the experience of a 1.7 m, 66.7 kg human wearing clothes (Rothfusz and Headquarters, 1990; Steadman, 1979). To the best of our knowledge, there is no comparable heat index for songbirds, much less songbird nestlings. We believe this is a notable gap in the study of songbird thermal physiology, and our current study underscores the need for increased scientific attention. To advance our understanding of the effects of intensifying heat, we need to be able to characterize the degree of heat that songbirds are experiencing.

### Implications and applications

As global temperatures warm, many animals are shifting their breeding ranges to higher latitudes or altitudes into cooler climates (Chen et al., 2011; Huang et al., 2023). However, some birds do not follow this pattern, and the tree swallow is one of these interesting exceptions (Mccaslin and Heath, 2020). In the last few decades, tree swallows along the eastern United States have expanded their breeding south into the hot and humid American Southeast (Shutler et al., 2012; Wright et al., 2019) – as far south as Alabama (Wright et al., 2019). There may be advantages to this change (e.g., better insect resources or reduced inter-specific competition for limited nesting cavities), but, for long-term success, the birds must have the physiological ability to cope with the environmental conditions of that area. Previous work across the continent has demonstrated that reproductive success was not sensitive to heatwaves in this species, even though other species showed concerning declines (Taff and Shipley, 2023). After mild heat exposure, tree swallow nestlings also showed some positive effects on body mass (Dawson et al., 2005; Shipley et al., 2022; Woodruff et al., 2023), a metric that predicts the likelihood of recruitment (Mccarty, 2001; Shipley et al., 2022). Adult tree swallows in the southern expansion range also exhibit more defensive aggression, have higher baseline corticosterone, and have a greater magnitude of stress-induced corticosterone compared to birds in the historic core of the range (Siefferman et al., 2023). Our new results add an important data point on an additional element of physiology that differs across latitudes in ways that should be adaptive, at least under current levels of climate change. Future applications of HSP90AA1 gene expression could include use in tracking long-term physiological adjustments to warmer climates and use in predicting species’ adaptive potential to future thermal challenges. Moving forward, we urge more researchers to take up this ‘sliding window’ approach applied to additional potential biomarkers sourced from reviews (e.g., aldosterone, Corbett et al., 2023; TRPV4, Sur and Sharma, 2025) and transcriptomic assays (e.g., NR4A3, PIK3CD, Woodruff et al., 2025). Coupled with among-population and among-species comparisons, we will be better equipped to predict and mitigate climate impacts on birds.

## Data Availability

The datasets presented in this study can be found in an online repository, found here: https://doi.org/10.5061/dryad.tdz08kq94.
