# Supplementary material for: Heat shock protein gene expression varies among populations but does not strongly track recent environmental conditions: implications for biomarker development
Source: Front Physiol. 2025 Oct 23;16:1601369. doi: 10.3389/fphys.2025.1601369 (PMC12588927; doi:10.3389/fphys.2025.1601369)
Supplement: Supplementary file 1 [file Supplementaryfile1.docx]

**Supplemental Information**

Contents

[A. Estimating Nestling Age & Nestling Sampling 1](#_Toc207975617)

[B. Environmental Condition Variables 1](#_Toc207975618)

[C. RNA Extraction, cDNA Synthesis, and Quantitative PCR 2](#_Toc207975619)

[D. Silding Window Analysis 3](#_Toc207975620)

[*Table S1* 3](#_Toc207975621)

[*Table S2* 3](#_Toc207975622)

[*Table S3* 3](#_Toc207975623)

[*Table S4* 4](#_Toc207975624)

[**Literature Cited** 5](#_Toc207975625)

## A. Estimating Nestling Age & Nestling Sampling

When hatch date was unknown, we measured several nestlings per nest and estimated nestling age (days-post-hatch) using growth data from Wolf et al. (2021) and McCarty (2001), which reported similar growth trajectories across 14 populations. Estimated-age compared to known-age D12 nestlings in Pennsylvania, Tennessee, North Carolina, and South Carolina, had statistically similar body mass (F_1,89_ = 0.90, p = 0.35). However, estimated-age nestlings had significantly shorter wing length (β = -6.27 ± 2.35 mm, F_1,89_ = 14.38, p = 0.003). At this age, tree swallow wings grow 4-6 mm per day (McCarty 2001), so a 6-7 mm difference in wing length likely amounts to, at most, a one day disparity between estimated-age and known-age D12 nestlings (i.e., D11 when estimated D12). We also found no effect of nestling mass or days-post-hatch on HSP90AA1 gene expression (mass: F_1,145_ = 0.93, p = 0.34; days-post-hatch: F_1,145_ = 2.43, p = 0.12). Because nestling thermoregulation begins around D6 (Marsh 1980), well before our sample collections, this potential difference between estimated-age versus known-age nestlings does not impact our aim of studying nestling thermal tolerance mechanisms.

Some nests from MA were part of a nest parasite study that treated nesting material (grass) with water (n = 8 nests), permethrin insecticide (n = 4 nests), or no treatment (n = 3 nests). There was no difference in HSP gene expression between these treatment groups (F_2,2_ = 1.25, p = 0.32), so we kept all nests in the study.

In North Carolina, a storm induced abnormally cold, rainy weather for three days. To maintain our focus on birds measured during typical ambient conditions, we excluded any nests that had already hatched during the cold snap. Nestlings that were sampled before the cold snap and those that hatched after the cold snap did not statistically differ in HSP gene expression (F_1,12_ = 0.31, p = 0.59).

## B. Environmental Condition Variables

Heat index formula sourced from Zulovich and DeShazer (1990) in which *Tdb* = dry bulb temperature in degrees Celsius and *Twb* = wet bulb temperature in degrees Celsius.

*Formula S1: HI = 0.6 Tdb + 0.4 Twb*

We extracted hourly dry and wet bulb temperatures from the closest weather station that reported these variables - Hartford Bradley International Airport, CT US 50 km south of the MA field site. To ensure that this weather station accurately reflected temperatures at the field site, we compared daily minimum and maximum temperatures between the Hartford Bradley International Airport and the Amherst, MA weather station located ~3 km from the field station (only daily dry bulb temperature reported by the Amherst station). There was no effect of date or weather station on minimum or maximum dry bulb temperature during our period of study (*Table S1*). Therefore, we moved forward with using the Hartford Bradley International Airport weather station in our analyses.

To estimate the upper limit of the Tree Swallow ‘comfort’ zone, we used two inferences. For one, other comparably sized songbirds reach the upper end of their thermoneutral zone at ~37.5°C (extracted from Appendix S1 in Wolf et al. 2017). In addition, prior experimental work found elevated HSP gene expression after four hours of temperatures exceeding about 38°C (Woodruff et al. 2025). This experiment also found that temperatures in the nest cup, where nestlings reside, were 12.3 ± 0.8°C warmer than the ambient environment (range of nest temperature elevation above ambient in control nests: 6-19°C; Woodruff et al. 2025).

C. RNA Extraction, cDNA Synthesis, and Quantitative PCR

We made the *a priori* decision to use the median mass nestling blood sample in our analysis, or the nestling with the closest mass to the median, if the median-mass nestling was not bled or if the sample volume was insufficient (<25 uL).

We used the phenol-chloroform-based Trizol method, following the manufacturer’s instructions to extract RNS from blood samples. Phase Lock Gel tubes (QuantaBio, Massachusetts, USA) were used to improve yield. Then, to analyze RNA quality and quantity, we resuspended total RNA in water and used an Epoch Microplate Spectrophotometer (Biotek, Vermont, USA).

Next, to synthesize cDNA, we treated 1 μg RNA with RNaseOUT Recombinant Ribonuclease Inhibitor (Thermo Fisher Scientific, Waltham, Massachusetts, USA) and DNase (Promega, Wisconsin, USA) for reverse-transcription using oligo dT primers and Superscript III (Invitrogen, California, USA).

To measure HSP gene expression we performed qPCR. We added 3 μl of 1:50 diluted cDNA, or 3 μl water for NTCs, and 7μl mix (1.94 μl water, 0.03 μl forward primer, 0.03 μl reverse primer, and 5 μl SYBR) into each well. We set the thermocycling condition to: 95°C for 10 min, 95°C for 30 s, 60°C for 30 s, and 70°C for 30 s. Finally, a dissociation phase (95°C for 1 min, 55°C for 30 s, and 95°C for 30 s) confirmed single-product specificity.

For 2 samples, RNA concentrations were low (< 110 ng/µL), so we equalized the amount of material loaded into the qPCR reaction by modifying our cDNA recipe and qPCR dilution. We used 300 ng of RNA for reverse transcriptase and later during qPCR, we used a 1:15 rather than 1:50 dilution qPCR to account for the lower concentration. All samples fell within the Ct range of our standard curves. Primers were previously validated in D12 blood serial dilutions (see *Table S3*). We ran cDNA in triplicate and averaged across replicates to produce a final gene expression value per sample. If one replicate did not fall within one Ct of the other two replicates, the outlying replicate was omitted. If all three replicates did not fall within one Ct of each other, the triplicate was re-run.

Our internal reference gene, MRPS25, was stably expressed across populations with a difference of less than one between mean Ct’s and was previously shown to be unaffected by experimental heat (Woodruff et al. 2023).

Nestlings were molecularly sexed as part of a previous study using these samples. We found no effect of sex, or the interaction between sex and state on HSP gene expression (Log_2_ Relative Quantity of HSP90AA1 ~ Sex: F_2,134_ = 1.81, p = 0.17; State: F_5,134_ = 2.12, p = 0.07; Sex*State: F_5,134_ = 0.82, p = 0.54), so we did not pursue this further.

D. Silding Window Analysis

We did not include pre-hatch windows in our analysis because mothers modulate their incubation in varying weather (Coe et al. 2015; Huggins 1941), and so pre-hatching egg temperatures may be more consistent compared to post-hatch nest temperatures. We also felt confident that we were not inadvertently omitting naturally-occurring heat events by focusing on post-hatch windows because the primary environmental challenge occurring during early Spring incubation is cold not heat (Shipley et al. 2020).

**Tables**

| Model | Model Term | β | Standard Error | t Ratio | Prob>\|t\| |
| --- | --- | --- | --- | --- | --- |
| Daily minimum temperature |  |  |  |  |  |
|  | Date | 6.12e-7 | 1.87e-6 | 0.33 | 0.75 |
|  | Station | -1.13 | 1.01 | -1.11 | 0.27 |
| Daily maximum temperature |  |  |  |  |  |
|  | Date | 2.09e-6 | 1.4e-6 | 1.49 | 0.15 |
|  | Station | 0.03 | 0.76 | 0.04 | 0.97 |

*Table S1: Models comparing temperatures recorded by two weather stations near our MA field site.*

| Gene Name | Primer Sequences | Efficiency | Citation |
| --- | --- | --- | --- |
| HSP90AA1 | FWD: GCTTCCAGAAGATGAGGAAGAG  RVS: GCAGCATGGAGAAGTGACTAA | 102% | (Woodruff et al. 2022) |
| MRPS25 | FWD: ATCACATCCAGCAACCTTTGG  RVS: CAGGGAACTTGGCCTTCAATC | 103% | (Woodruff et al. 2022) |

*Table S2*: *Primer sequences, efficiencies, and citations.*

*Table S3 and S4 included in included spreadsheet*

*Table S3: Full sliding window model output. AICc values relative to the null model (ΔAICc), beta estimate effect sizes (β), standard error (SE), and model intercept are reported. Window openings represent time furthest from sampling and window closures represent time nearest to sampling.*

*Table S4: Full sliding window model output using just samples collected in South Carolina. AICc values relative to the null model (ΔAICc), beta estimate effect sizes (β), standard error (SE), and model intercept are reported. Window openings represent time furthest from sampling and window closures represent time nearest to sampling.*

## **Literature Cited**

Marsh, R. L. (1980). Development of temperature regulation in nestling tree swallows. *The Condor, 82*(4), 461.

McCarty, J. P. (2001). Variation in growth of nestling tree swallows across multiple temporal and spatial scales. *The Auk, 118*(1), 176.

Wolf, S. E., Stansberry, K. R., Content, K. R., & Rosvall, K. A. (2021). A putative telomerase activator has tissue-specific effects on telomere length in a developing songbird. *Journal of Avian Biology, 52*(2).

Woodruff, M. J., Zimmer, C., Ardia, D. R., Vitousek, M. N., & Rosvall, K. A. (2022). Heat shock protein gene expression varies among tissues and populations in free living birds. *Ornithology, 139*(3), ukac018.

Zulovich, J., & DeShazer, J. (1990). Estimating egg production declines at high environmental temperatures and humidities.
